# Antidepressant effect of transcranial pulse stimulation (TPS) targeting neuropsychiatric disorders: a retrospective analysis

**DOI:** 10.1017/S0033291726103274

**Published:** 2026-02-06

**Authors:** Michael Mitterwallner, Sonja Radjenovic, Daria Grigoryeva, Lena Bender, Martin Gaal, Sarah Osou, Anna A. Zettl, Nina Plischek, Patrick Lachmair, Katrin Herzhauser, Eva Matt, Roland Beisteiner

**Affiliations:** 1 Department of Neurology, Medical University of Vienna, Vienna, Austria; 2 Department of Pediatrics and Adolescent Medicine, Medical University of Vienna, Vienna, Austria

**Keywords:** depression, neuromodulation, noninvasive brain stimulation, transcranial pulse stimulation, ultrasound

## Abstract

**Background:**

Depression is a common comorbidity in neuropsychiatric disorders, affecting a significant proportion of patients with neurodegenerative diseases. Traditional antidepressants show limited efficacy, particularly in cases involving comorbid depressive symptoms, highlighting the need for alternative treatments.

**Methods:**

Here we provide the first data on possible benefits of add-on therapy with transcranial pulse stimulation (TPS). Based on the largest patient sample in the emerging field of focused ultrasound (FUS) neuromodulation to date, a retrospective analysis was conducted on 88 patients with various neuropsychiatric diagnoses to evaluate the impact of TPS on depressive symptoms, measured by the Beck Depression Inventory (BDI-II).

**Results:**

The study revealed significant improvements in BDI-II scores posttreatment (*N* = 88), with the most substantial effects observed in more severely impacted patients: individuals with minimal to severe depression (BDI-II ≥9; *N* = 32) experienced an average reduction of 5.22 points (29.46%), while those with mild to severe depression (BDI-II ≥14; *N* = 15) showed an even greater mean improvement of 10.40 points (40.51%). These results surpassed established thresholds for clinical relevance and substantially exceeded placebo effect sizes observed in relevant brain stimulation studies. Moreover, depression score improvement was independent of diagnostic group (dementia, movement disorders, or other), improvement of the primary diagnosis, antidepressant medication, and baseline cognitive status, highlighting the potential of TPS as an effective therapeutic add-on intervention for patients receiving state-of-the-art treatments.

**Conclusions:**

The study’s findings indicate that TPS enhances depression outcomes in neuropsychiatric patients, particularly in those with more severe depressive symptoms.

## Introduction

Depression as a comorbidity for a wide range of neuropsychiatric diseases is of high practical therapeutic relevance. Depressive symptoms affect up to 40% of patients with mild cognitive impairment (MCI) (Ismail et al., 2017), 42% of those with Alzheimer’s disease (AD) (Zhao et al., 2016), 50% of patients with Parkinson’s disease (PD) (Tian, Kang, Liu, & Yu, 2022), and 34% of stroke patients (Mitchell et al., 2017).

Effects of antidepressant medication are limited. Second-generation antidepressants, the most commonly prescribed for major depressive disorder, fail to elicit a response in 40% of patients and do not achieve remission in 70% (Gartlehner et al., 2011). While the benefit of antidepressants over placebo increases with depression severity, it is minimal or nonexistent in patients with mild to moderate symptoms (Fournier et al., 2010). In the context of comorbid depression, antidepressants show no statistically significant advantage over placebo in treating depression in AD (Orgeta, Tabet, Nilforooshan, & Howard, 2017) and unstable results in PD (Rocha, Murad, Stumpf, Hara, & Fuzikawa, 2013). Moreover, they increase the risk of serious and non-serious adverse events (Jakobsen, Gluud, & Kirsch, 2020).

Noninvasive brain stimulation (NIBS) is a promising therapy for depressive symptoms. Electrical and electromagnetic technologies, such as transcranial magnetic stimulation (TMS) and transcranial direct current stimulation (tDCS), have been effective in treating comorbid depression and depressive mood in Parkinson’s disease (Zheng et al., 2022) and poststroke depression (Yanyu, Ying, Kexin, & Jin, 2023). While these techniques did not significantly improve depression in AD and MCI, they were effective in alleviating pooled neuropsychiatric symptoms in AD patients (Teselink et al., 2021). Moreover, NIBS in combination with antidepressant medication (Tao et al., 2024) or psychosocial interventions (He et al., 2022) was found to be substantially more effective in reducing depressive symptoms than either treatment alone. Consequently, NIBS has the potential to enhance the outcomes for patients already on state-of-the-art therapy.

A very novel development in noninvasive brain stimulation is neuromodulation with focused ultrasound (FUS), which currently attracts considerable attention and shows exponential publication growth (Matt, Radjenovic, Mitterwallner, & Beisteiner, 2024; Pellow, Pichardo, & Pike, 2024; Sarica et al., 2022). A large variability of FUS neuromodulation technologies are currently used applying mono-/multifrequency and single-/multitransducer approaches (Pellow et al., 2024; Qin et al., 2024; Sarica et al., 2022). Most clinical neuromodulation studies have been performed with single-transducer techniques (Matt et al., 2024). In contrast to long-established electrical and electromagnetic techniques, ultrasound neuromodulation offers specific advantages in a therapeutic setting: (1) brain pathology does not alter the target for stimulation as possible with electromagnetic field distortions (Mantell et al., 2021), (2) noninvasive modulation of deep brain areas is possible (Badran et al., 2020; Legon, Ai, Bansal, & Mueller, 2018), and (3) the stimulation is more focal (Truong, Thomas, Hampstead, & Datta, 2022).

The very first patient study using transcranial pulse stimulation (TPS) indicated that this approach may improve depressive symptoms in Alzheimer’s disease (Beisteiner et al., 2019). Within the broader FUS neuromodulation landscape, TPS represents a navigated nonlinear multifrequency single-transducer implementation. It is based on shockwave technology and delivers ultrashort (≈3 μs) pulses in the ultrasonic frequency range with high peak pressure and low energy flux density (Beisteiner, Hallett, & Lozano, 2023). Notably, ultrashort pulse durations (μs range) and high peak pressures (>30 MPa) are not unique to TPS, but can also be realized in monofrequency as well as multifrequency ultrasound techniques (Radjenovic, Dörl, Gaal, & Beisteiner, 2022; Weinreb & Moses, 2022). Subsequent TPS work, including a cross-over RCT (Matt, Mitterwallner, et al., 2025), has confirmed antidepressant effects in Alzheimer’s disease (Matt, Dörl, & Beisteiner, 2022) and in major depressive disorder (Cheung et al., 2023). In parallel, different monofrequency FUS neuromodulation implementations (tFUS) – using continuous or burst sinusoidal ultrasound waveforms at lower peak pressures – have also demonstrated antidepressant effects, often in pilot and early-phase studies (Attali et al., 2025; Barksdale et al., 2025; Fan et al., 2024; Riis, Feldman, Losser, Mickey, & Kubanek, 2024; Riis et al., 2023; Schachtner et al., 2024) and in sham-controlled RCTs (Cai et al., 2025; Oh et al., 2024; Reznik et al., 2020; Riis et al., 2025). However, for assessing the value of TPS as a novel add-on therapy, clinical effects in complex neuropsychiatric real-life settings are important.

Here, we provide a first retrospective analysis on this topic in patients on state-of-the-art treatments for various neuropsychiatric diseases. Specifically, our objective was to evaluate the efficacy of TPS in treating comorbid depressive symptoms across varying baseline severity levels in neuropsychiatric patients. Although this is a retrospective analysis, we formulated the following a priori assumptions based on previous findings on NIBS and depression: (1) TPS treatment would lead to a reduction in depressive symptoms across the full sample, and (2) patients with higher baseline depression severity would show more pronounced improvements. These hypotheses were tested using within-subject comparisons and subgroup analyses. In addition, we conducted an exploratory linear mixed model analysis to investigate whether primary diagnosis, improvement of the primary condition, and antidepressant medication influenced changes in depressive symptoms. Using correlation analyses, we further examined whether baseline cognitive status was associated with treatment-related changes in self-reported depression. The study represents the largest clinical TPS cohort currently available and one of the largest datasets within the emerging field of focused ultrasound neuromodulation, providing a unique perspective on treatment effects in real-world clinical settings.

## Methods

### Sample

The initial sample comprised patients with preexisting, externally established neuropsychiatric diagnoses who requested and self-funded a TPS treatment trial at the TPS Therapy and Development Center, and were assessed for depressive symptoms using the Beck Depression Inventory (BDI-II). All patients were treated for their primary diagnosis, with Alzheimer’s disease, Parkinsonian syndrome, dementia, mixed dementia, and vascular dementia being the most common conditions (see Supplement S1). Patients were excluded from the study if they did not provide sufficient BDI-II data or did not self-report on the BDI-II questionnaire. To ensure data comparability, repeated treatment cycles (i.e. subsequent visits) were also excluded, yielding a sample of 88 patients (male: 51; female: 37; age range: 28–84; mean age: 70.31 ± 9.77). As of now, this is the largest patient sample treated with TPS and one of the largest clinical cohorts reported within the broader field of FUS (Matt et al., 2024). Additionally, utilizing subsets of the entire sample, focused analyses of 32 more impacted patients (male: 19; female: 13; age range: 28–84; mean age: 70.09 ± 10.90) and 15 severely impacted patients (male: 9; female: 6; age range: 28–82; mean age: 68.20 ± 13.24) could be conducted (see section ‘Data-analytical strategy’ for further details). Some of the data reported here were previously analyzed in the context of primary dementia and Parkinson’s disease diagnoses (Osou et al., 2023; Radjenovic et al., 2025); however, this study presents novel findings on depressive symptoms.

### Study design

This open-label, uncontrolled, retrospective study involved patients undergoing transcranial pulse stimulation (TPS) therapy using the NEUROLITH TPS system (Storz Medical AG, Tägerwilen, Switzerland) (Beisteiner et al., 2023, 2019) as an add-on therapy or therapeutic attempt at the TPS Therapy and Development Center in Vienna, Austria. The study was submitted to the Ethics Committee of the Medical University of Vienna and received approval (reference number: 1821/2021).

Potential contraindications, such as thrombosis, pregnancy, tumors in the treatment area, cortisone treatments within 6 weeks prior to the first application, and metal objects in the head were assessed along with the patients’ medical histories. To exclude contraindications and to develop individualized TPS treatment plans, high-resolution magnetic resonance imaging (MRI) was performed externally shortly before the start of treatment. Standard non-TPS treatment was individually optimized by the patients’ external treating neurologists or psychiatrists and remained unchanged throughout the therapy phase to eliminate secondary influences on TPS effects. At baseline, 20 patients (22.7% of 88) were on classical antidepressants (SSRI/SNRI/NDRI/NaSSA/SARI). All medication regimens remained stable throughout the entire TPS treatment period (see Supplement S1). Prior to therapy, patients provided informed consent.

TPS is a tool for personalized precision medicine and requires comprehensive clarification of the patients clinical and neuropathological situation to allow optimum treatment settings. Following current recommendations (Beisteiner, Lozano, Di Lazzaro, George, & Hallett, 2024), therapy was highly individualized with a duration ranging from 2 to 4 weeks, comprising 8 to 12 sessions, each lasting 30 to 45 minutes. Stimulation parameters were individualized as well, with the most common settings involving 4000 ultrashort ultrasound pressure pulses (approximately 3 μs) per session (min/max: 3200–4300), an energy flux density of 0.25 mJ/mm^2^ (min/max: 0.10–0.25 mJ/mm^2^), and a pulse repetition rate of 4 Hz. Treatment protocols were carefully tailored to each patient’s clinical status, symptom profile, and underlying brain pathology (Beisteiner et al., 2024). The selection of target brain regions was guided by Beisteiner et al. (2019) for patients with dementia and by a protocol developed by Matt, Plischek, et al. (2025) for patients with movement disorders. Stimulation of the left dorsolateral prefrontal cortex (DLPFC) (classical brain stimulation target for mood improvement) was consistently applied in all but one case. In this single patient, the stimulation protocol encompassed the anterior cingulate cortex (ACC), a key region in the salience network associated with depressive disorders (Dunlop, Hanlon, & Downar, 2017). In addition to the DLPFC, several other regions were frequently targeted in this study’s patient cohort, including the precuneus, bilateral parietal cortex, anterior cingulate cortex, temporal cortex, primary motor cortex, and supplementary motor area (see Supplement S1 for details). Furthermore, patients completed the self-reported Beck Depression Inventory (BDI-II) a few days before and after therapy.

Our research question aimed to determine whether the retrospective data from our real life individualized TPS treatment settings indicated improvement for depression scores in neuropsychiatric disorders.

### Measures

*Beck Depression Inventory (BDI-II)*

The primary outcome of this study was assessed using the Beck Depression Inventory (BDI-II) (Beck, Steer, & Brown, 1996), a revised version of the original BDI. The inventory comprises 21 items, each rated on a severity scale from 0 to 3. Patients are instructed to reflect on their experiences over the 2 weeks preceding the questionnaire. The items assess symptoms such as sadness, pessimism, loss of pleasure, guilt, suicidal ideation, irritability, loss of energy, and fatigue. Scoring is categorized as follows: 0–8 indicates no depression; 9–13 indicates minimal depression; 14–19 indicates mild depression; 20–28 indicates moderate depression; and 29–63 indicates severe depression.

*Additional measures for linear mixed models*

To investigate whether improvement of primary diagnosis influenced depression score improvement, the following measures were included:

1. Consortium to Establish a Registry for Alzheimer’s Disease (CERAD) corrected total score (CTS) – a measure of cognitive deficits associated with dementia (Chandler et al., 2005; Fillenbaum & Mohs, 2023).
2. Unified Parkinson’s Disease Rating Scale (UPDRS) total score – an assessment of overall disease severity in Parkinson’s disease, including motor and nonmotor symptoms (Movement Disorder Society Task Force on Rating Scales for Parkinson’s Disease, 2003).
3. Unified Parkinson’s Disease Rating Scale (UPDRS-III) – a subscale specifically measuring motor impairment in Parkinson’s disease (Movement Disorder Society Task Force on Rating Scales for Parkinson’s Disease, 2003).
4. Montreal Cognitive Assessment (MoCA) – a screening tool for mild cognitive impairment (Nasreddine et al., 2005).
5. Mini-Mental State Examination (MMSE) – a test for global cognitive function (Tombaugh & McIntyre, 1992).

*Patient safety evaluation and adverse events*

Patients were asked to report any adverse events using an open-format protocol. These statements were classified according to the Common Terminology Criteria for Adverse Events (CTCAE, v. 5.0).

### Data-analytical strategy

The statistical methods included paired *t* tests or Wilcoxon signed rank tests to compare pre- and posttreatment BDI-II scores, linear mixed model (LMM) analysis, correlation analyses between baseline cognitive status (MMSE or MoCA) and BDI-II change scores, and descriptive statistics for worsening of BDI-II scores as well as safety and adverse events.

First, a pre-/postcomparison was conducted for the entire sample (*N* = 88). Subsequently, refined analyses were performed on subsets of 32 and 15 patients with BDI-II scores of ≥9 and ≥14 respectively, thereby excluding individuals with no depression (scores 0–8) in the second and individuals with no and minimal depression (scores 0–13) in the third analysis and thus incrementally focusing on more severely impacted subsets. The first subsample (*N* = 32) was defined according to BDI-II classification, which considers scores of ≥9 as indicative of clinically relevant depressive symptoms. The second subsample (*N* = 15) followed the criteria set by Hautzinger, Keller, and Kühner (2006), which classifies BDI-II scores of ≥14 as reflecting clinically relevant depression. Overall, this stepwise approach was based on literature evidence that patients with higher initial depression scores would show more pronounced improvements after therapy (Fournier et al., 2010).

In terms of assumptions, significant outliers (as indicated by boxplots) were found in the differences between paired values within the entire sample (*N* = 88), necessitating the use of a Wilcoxon signed rank test. The second analysis (*N* = 32) revealed a non-normal distribution of the difference scores, as indicated by the Shapiro–Wilk test (*p* < 0.05). However, since the sample size exceeded *N* = 30, the assumption of normality was not required (Stone, 2010), and a paired *t* test was conducted. The third analysis (*N* = 15) met all statistical requirements, once again allowing for a paired *t* test. Since all three tests were conducted on the same dataset to examine the same primary effect (pre- vs. posttreatment change), *p* values were adjusted using the Bonferroni–Holm correction. Mean BDI-II change was considered clinically meaningful if it exceeded the anchor-based threshold of 5 points (Hiroe et al., 2005) and represented a reduction of at least 17.5% from baseline (Button et al., 2015). Pre-/postcomparisons were conducted using SPSS 29.0, applying a significance threshold of *α* = .05. Plots were generated with JASP 0.19.0.

Furthermore, the influence of primary diagnosis, improvement of primary diagnosis, and antidepressant medication on BDI improvement was assessed. To this end, patients were categorized into three primary diagnosis groups: dementia (*N* = 56), movement disorders (*N* = 24), and a third group comprising diagnoses other than dementia or movement disorders, referred to as ‘other’ (*N* = 8) (see Supplement S1). Improvement of the primary diagnosis was assessed using CERAD CTS change scores for the dementia and ‘other’ groups and UPDRS-III change scores for the movement disorder group. These change scores were calculated by subtracting pretreatment from posttreatment scores, or vice versa, depending on the scale’s directionality, ensuring that positive values consistently indicated improvement. To maximize sample inclusion, alternative measures were used when primary diagnosis improvement data were unavailable: MoCA change scores for two dementia patients, MMSE change scores for one dementia patient, and UPDRS total score change scores for two movement disorder patients. Three patients were excluded due to insufficient data. All scores were *z* transformed for comparability across measures. Antidepressant medication status was coded as a binary variable (0 = no, 1 = yes).

Given the unequal distribution of patients across groups, a series of linear mixed effects models (LMM) was conducted (Baayen, Davidson, & Bates, 2008). To address the skewed distribution of BDI pre- and posttreatment scores, a logarithmic transformation was applied to approximate normality. Fixed effects for *time* (indicating treatment effects), *group* (indicating primary diagnosis), *primary scores* (indicating improvement of primary diagnosis), and *antidepressant medication* (yes/no) were sequentially introduced, along with their interactions. *Antidepressant medication* was only allowed to interact with *time*, and therefore no four-way interaction was included in the model. Participant ID was modeled as a random intercept to account for individual variability.

All LMM analyses were conducted in R (version 4.2.2) (R Core Team, 2022) and RStudio (version 2023.06.1+524) (RStudio Team, 2022) using the nlme package (version 3.1-160) (Pinheiro J, Bates D, DebRoy S, Sarkar D, R Core Team, 2022). Model comparisons were performed using maximum likelihood (ML) to evaluate whether the inclusion of each predictor significantly improved model fit. The contribution of fixed effects was assessed using likelihood-ratio tests (LRT), as implemented in the anova() function. Akaike information criterion (AIC) and Bayesian information criterion (BIC) were used as additional criteria, with lower values indicating relatively better model fit while accounting for model complexity. Once the final model was selected based on LRTs using ML, it was re-estimated using restricted maximum likelihood (REML) to obtain unbiased variance estimates. Statistical significance was set at *α* = .05. The final model retained *time* as a significant predictor of logBDI scores, while *group*, *primary score*, and *antidepressant medication* were not significant and were excluded: logBDI ∼ time + (1|ID). The linear mixed model addressed a distinct research question and was therefore not included in the multiple testing correction.

Given that the majority of the cohort had dementia (*N* = 56) or movement disorders including Parkinson’s disease (*N* = 24), we examined whether baseline cognitive status was associated with changes in depressive symptoms. Specifically, we computed Spearman’s rank correlations between baseline MMSE and BDI-II change scores (*N* = 67), and between baseline MoCA and BDI-II change scores (*N* = 60) (BDI-II change calculated as pre − post). MMSE or MoCA assessments were available primarily, though not exclusively, for patients with dementia, and therefore these analyses were conducted in the available subsamples rather than in the full sample. Nonparametric correlations were chosen because BDI-II change scores contained outliers (as indicated by boxplots). These bivariate analyses were performed separately from the linear mixed effects models to minimize collinearity and redundancy, as the LMM already included *z*-standardized change scores derived from the same cognitive measures to index improvement in the primary diagnosis. Correlational analyses were performed in SPSS 29.0 with a significance threshold of *α* = .05. As these analyses addressed an exploratory question, they were not subjected to multiple testing correction.

Concerning patient safety and adverse events, the most common side effects were identified.

## Results

**Figure 1.**
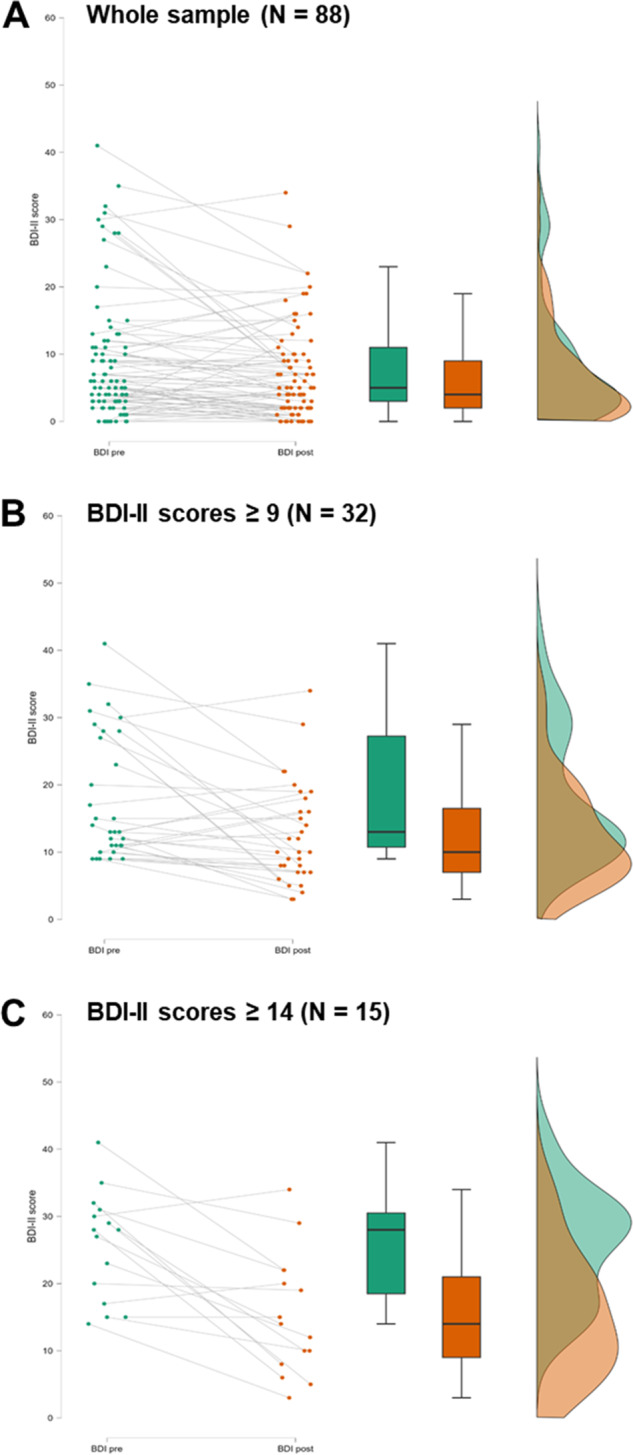
Analyses results. (A) Results for the whole sample (*N* = 88): BDI-II scores were significantly reduced from pre- to posttreatment (two-sided Wilcoxon test, *p* = .009). (B) Results for patients with minimal to severe depression scores (BDI-II scores ≥9, *N* = 32): BDI-II scores were significantly reduced from pre- to posttreatment (two-sided paired *t* test, *p* = .002). (C) Results for patients with mild to severe depression scores (BDI-II scores ≥ 14, *N* = 15): BDI-II scores were significantly reduced from pre- to posttreatment (two-sided paired *t* test, *p* < .001). Each figure (A, B, C) displays individual patient data points, with green points representing pretreatment scores and orange points representing posttreatment scores. The centered boxplots show the pre- and posttreatment data, while the raincloud plots illustrate the distribution of scores, with green indicating the baseline and orange indicating the postintervention distribution.

The Wilcoxon signed rank test for the entire sample (*N* = 88) indicated a significant difference between the two assessments, with the median score before treatment (Mdn = 5.00) being higher than the median score after treatment (Mdn = 4.00). The Wilcoxon test yielded a standardized test statistic of *z* = −2.63, with a corresponding *p* = .009 (two-tailed) and *r* = .28, revealing a small-to-moderate effect. Descriptively comparing the means revealed a change of 1.93 between pre- and posttreatment scores, corresponding to a 22.73% improvement compared to baseline (Figure 1A).

Refining the analysis for the more impacted patients (BDI-II scores ≥9, *N* = 32), a paired *t* test was performed to compare mean scores before treatment (17.72 ± 9.39) and after treatment (12.50 ± 7.45). The analysis demonstrated a significant mean improvement of 5.22 points from pre- to posttreatment (*t*(31) = 3.42, *p* = .002 (two-tailed); Figure 1B) (29.46% improvement compared to baseline), with a 95% confidence interval for the mean difference ranging from 2.11 to 8.33. Cohen’s *d* (0.60) revealed a moderate effect.

Further refining the analysis for the severely impacted patients (BDI-II scores ≥14, *N* = 15), the paired *t* test demonstrated a significant mean improvement of 10.40 points (*t*(14) = 4.21, *p* < .001 (two-tailed)) (40.51% improvement compared to baseline) when pre- (25.67 ± 8.07) and posttreatment scores (15.27 ± 8.98) were compared (Figure 1C). Cohen’s *d* was 1.09, denoting a strong effect, with the 95% confidence interval for the mean difference ranging from 5.11 to 15.69.

Following Bonferroni–Holm correction for multiple comparisons, all results remained statistically significant (uncorrected *p* values: .009, .002, < .001; corrected *p* values: .009, .004, .003).

Concerning the LMM results, the final model retained *time* as a significant predictor of logBDI scores, χ^2^(1) = 7.04, *p* = .008, while *group*, *primary score*, and *antidepressant medication* did not significantly contribute to model fit and were excluded. This suggests that treatment effects, represented by time, influenced logBDI scores, whereas primary diagnosis, improvement of primary diagnosis, and antidepressant medication had no statistically significant impact.

Baseline cognitive status was not associated with change in depressive symptoms: MMSE versus BDI-II change, *ρ* = 0.013, *p* = .917 (*N* = 67); MoCA versus BDI-II change, *ρ* = 0.117, *p* = .375 (*N* = 60). Within the limits of the available subsamples, we therefore found no evidence that more cognitively impaired patients reported different treatment-related changes in self-reported depression.

Of 88, 19 (21.6%) patients experienced minor BDI-II increases (<5 points, not clinically meaningful), while only 6 (6.8%) patients met the threshold for clinically meaningful worsening (increase ≥5 points).

Regarding patient safety and adverse events, 32.95% (*N* = 29) of the 88 patients reported fatigue, 30.68% (*N* = 27) stated pain sensations, and 22.73% (*N* = 20) experienced dizziness. When all 890 treatment sessions were considered, side effects occurred in 19.89% (177 sessions) of the TPS sessions, with fatigue (6.40%) (57 sessions), pain (4.94%) (44 sessions), and dizziness (4.04%) (36 session) being the most common. All side effects resolved within 24 hours, and no major adverse events were observed.

## Discussion

This study explored the efficacy of transcranial pulse stimulation (TPS) – a navigated multifrequency single-transducer FUS neuromodulation technique –as an add-on therapy for depressive symptoms in patients with various neuropsychiatric disorders as their primary diagnosis. Within current literature, this is the largest patient sample treated with TPS and one of the largest clinical cohorts reported within the broader field of focused ultrasound neuromodulation (Matt et al., 2024).

The analysis demonstrated statistically and clinically significant improvements in depression symptoms following TPS treatment across varying degrees of baseline depression severity. In the entire sample (*N* = 88), a significant reduction in BDI-II scores was observed, indicating a general trend toward symptom improvement (22.73% improvement compared to baseline). More notably, patients with higher baseline depression scores exhibited greater therapeutic benefits (compare Fournier et al., 2010). Specifically, individuals with minimal to severe depression (BDI-II ≥9; *N* = 32) experienced an average reduction of 5.22 points (29.46% improvement compared to baseline), while those with mild to severe depression (BDI-II ≥14; *N* = 15) showed an even greater mean improvement of 10.40 points (40.51% improvement compared to baseline). These focused results not only achieved statistical significance, but also surpassed the established clinical relevance threshold of 5 BDI-II points (Hiroe et al., 2005) and a reduction of at least 17.5% from baseline (Button et al., 2015), suggesting that TPS may effectively ameliorate depressive symptoms in patients with neuropsychiatric conditions. In the absence of sham control, and given our study design, clinical relevant deterioration in a small subset of patients (*N* = 6) likely reflects symptom fluctuation or expectancy effects rather than direct adverse effects of TPS. Nonetheless, the low incidence of clinically relevant worsening (6.8%) is reassuring regarding safety. Additionally, linear mixed model analysis indicated that depressive symptom improvement was independent of the primary diagnosis, improvement in the primary diagnosis, and antidepressant medication. Correlation analyses further showed that improvement was also independent of baseline cognitive status (MMSE and MoCA). Overall, TPS was generally well tolerated, with all side effects resolving within 24 hours and no major adverse events reported.

Furthermore, in the absence of a control group, it is important to consider potential placebo effects as previously described in NIBS literature (De Smet et al., 2021; Osou et al., 2023; Razza et al., 2018). To contextualize the findings, we therefore compared our TPS treatment effect size (Hedges’ *g* = 1.03) specifically with sham effect sizes reported in brain stimulation research. For this, we used the most severely impacted subsample with mild to severe depression (BDI-II ≥14; *N* = 15) as a benchmark for comparison since it represents the group of patients most likely to be diagnosed with comorbid depression, making the assessment of efficacy particularly relevant in this case. Given that the majority of our severely impacted depression patients had dementia or movement disorders (12 of 15), we examined sham-controlled NIBS studies focusing these primary conditions while also acquiring depression self-rating data. These criteria were defined to ensure an appropriate standard of comparison.

Within the emerging TPS literature, the sham arms of two randomized controlled TPS trials in Alzheimer’s disease and Parkinson’s disease provide the most directly comparable benchmarks, showing only small sham-related changes in BDI-II scores (Hedges’ *g* = 0.14 and 0.26, respectively; Matt, Mitterwallner, et al., 2025; Matt, Plischek, et al., 2025). Furthermore, Esposito et al. (2022) conducted a study using rTMS to alleviate MCI in 27 patients, with 16 assigned to the sham group. Unfortunately, the absence of relevant data precludes the calculation of Hedges’ *g* and limits our analysis to a descriptive comparison. While the study reported a median change of 3 between baseline and posttreatment (pre: 12, post: 9), our subsample with mild to severe depression (BDI-II ≥14; *N* = 15) demonstrated a more substantial median improvement of 10 points (median pre: 27.5, median post: 14.5), considerably exceeding the placebo group’s results in the prior study. As no additional research obtaining BDI-II scores was found, we broadened our search to include studies employing alternative depression self-assessment tools for comparative analysis. Among patients with mild to moderate dementia undergoing rTMS, the placebo effect related to depression was minimal (Hedges’ *g* = 0.04), as determined by the Geriatric Depression Scale (Ahmed, Darwish, Khedr, El Serogy, & Ali, 2012). We also identified NIBS studies that utilized the BDI-I, all focusing on Parkinson’s disease. Hedges’ *g* for the placebo effect size was *g* = 0.68 (Benninger et al., 2010) and *g* = 0.33 (Ferrucci et al., 2016) in tDCS research, *g* = 0.23 (Pal, Nagy, Aschermann, Balazs, & Kovacs, 2010) and *g* = 0.97 (Benninger et al., 2012) in rTMS studies, and *g* = −0.24 in an intermittent theta-burst stimulation trial (Benninger et al., 2011), indicating deterioration in the sham group. Finally, in a descriptive comparison, our mean treatment reduction of 40.51% compared favorably to the BDI-I improvement of 1.28% in a tDCS sham group (Doruk, Gray, Bravo, Pascual-Leone, & Fregni, 2014).

Comparing these sham effect sizes (Hedges’ *g* = 0.14, 0.26, 0.04, 0.68, 0.33, 0.23, 0.97, −0.24) and their mean (Hedges’ *g* = 0.30) with our treatment effect size (Hedges’ *g* = 1.03) provides further evidence for a meaningful effect. Overall, considering that our effect size surpasses the best available sham benchmark – NIBS trials in dementia and movement disorder patients with self-reported depression data – this finding highlights the therapeutic potential of TPS.

The efficacy of NIBS techniques, such as TMS and tDCS, has been previously documented in treating depression, particularly within the context of PD and poststroke conditions (Yanyu et al., 2023; Zheng et al., 2022). However, their effectiveness in addressing depressive symptoms associated with AD and MCI remains inconclusive (Teselink et al., 2021). Within this landscape, transcranial focused ultrasound stimulation has emerged as a novel NIBS method. FUS neuromodulation encompasses a range of ultrasound implementations, including mono- and multifrequency as well as single- and multitransducer approaches (Pellow et al., 2024; Qin et al., 2024; Sarica et al., 2022). TPS, the only nonlinear multifrequency form of neuromodulation, shares several practical advantages that have also been highlighted for linear monofrequency tFUS, including the possibility of deeper and more focal brain stimulation and reduced susceptibility to brain pathology (Badran et al., 2020; Legon et al., 2018; Mantell et al., 2021; Truong et al., 2022). These features make ultrasound neuromodulation a well-suited approach for alleviating depressive symptoms, and early clinical studies have already reported improvements. Our findings are consistent with emerging clinical evidence that TPS can ameliorate depressive symptoms in Alzheimer’s disease and in patients with clinically relevant depression (Beisteiner et al., 2019; Cheung et al., 2023; Matt, Mitterwallner, et al., 2025) and complement data on tFUS-based neuromodulation in depression (Attali et al., 2025; Barksdale et al., 2025; Cai et al., 2025; Fan et al., 2024; Oh et al., 2024; Reznik et al., 2020; T. Riis et al., 2024; T. S. Riis et al., 2025, 2023; Schachtner et al., 2024). This study contributes to this growing body of literature by demonstrating TPS efficacy for depressive symptoms across a broader range of neuropsychiatric conditions in a real-world clinical setting.

### Limitations

This study has several limitations that should be acknowledged. First, as with all open-label studies, the absence of a control group hinders the definitive attribution of observed improvements solely to the intervention, as potential placebo effects cannot be ruled out. However, comparing the effect size of this study with placebo effect sizes of randomized controlled trials solidly supports the efficacy of TPS. While we deliberately selected sham-controlled NIBS studies in dementia or movement disorder populations with self-reported depression data to ensure an appropriate standard of comparison, differences in stimulation parameters, devices, and sociodemographic characteristics between studies may still limit the direct comparability of these findings. Additionally, the open-label and retrospective design may introduce biases, such as the potential for selection bias and the influence of patients’ expectations on self-reported outcomes (Osou et al., 2023). Furthermore, the heterogeneity of the sample, which included various neuropsychiatric disorders, limits the generalizability of the findings to specific patient populations. Nevertheless, the linear mixed model analyses demonstrated depressive symptom reduction independent of the diagnostic group. Finally, the use of BDI-II as the sole measure of depressive symptoms, while widely validated, may not capture the full complexity of depression in neuropsychiatric contexts. Future research should include controlled, randomized trials with larger and more homogeneous subsamples to confirm these findings and further explore the potential of TPS and related forms of FUS neuromodulation in treating depressive symptoms.

## Conclusion

In summary, our study indicates that TPS therapy offers a promising new avenue for treating comorbid depressive symptoms in neuropsychiatric disorders, particularly for patients with more severe symptoms. Despite the limitations inherent to the study design, the effect sizes observed, combined with comparative analyses against sham-controlled trials, support the efficacy of TPS as an add-on therapy. Further research, ideally involving randomized controlled trials, is warranted to establish its role in clinical practice.

## Supporting information

10.1017/S0033291726103274.sm001 Mitterwallner et al. supplementary material Mitterwallner et al. supplementary material

## Supplementary material

The supplementary material for this article can be found at http://doi.org/10.1017/S0033291726103274.
