# Supplementary material for: Antidepressant effect of transcranial pulse stimulation (TPS) targeting neuropsychiatric disorders: a retrospective analysis
Source: Psychol Med. 2026 Feb 6;56:e40. doi: 10.1017/S0033291726103274 (PMC12902169; doi:10.1017/S0033291726103274)
Supplement: Mitterwallner et al. supplementary material [file S0033291726103274sup001.docx]

Supporting Information Table S1

*Patients’ characteristics, medication, stimulated brain regions, and individual BDI-II-Scores. (ext. = extended, r = right, l = left)*

| **ID** | **Gender** | **Age** | **Main diagnosis** | **Diagnosis group** | **Medication (antidepressants are shown in bold)** | **Stimulated Brain Regions (numbers in columns indicate pulse counts for left DLPFC-including ROI)** | **BDI-II pre** | **BDI-II post** |
| --- | --- | --- | --- | --- | --- | --- | --- | --- |
| P01 | f | 61 | Alzheimers's Disease | 1 | Rivastigmin 13.3mg/24h | frontal bilateral (700), parietal bilateral, precuneus, occ/temp bilateral | 0 | 0 |
| P02 | m | 68 | Alzheimers's Disease | 1 | Atorvasstatine 10 mg, Memantine 20mg, Rivastigmin 13.3 mg/24h, **Trazodone 100mg** | frontal bilateral (500), parietal bilateral, precuneus, occ/temp bilateral | 15 | 10 |
| P03 | f | 75 | Alzheimers's Disease | 1 | Candeblo 8mg, Nimvastid 6mg, Oleovit D3, Thrombo Ass 100mg | frontal bilateral (600), parietal bilateral, precuneus, anterior cingulum | 2 | 2 |
| P04 | f | 68 | Alzheimers's Disease | 1 | Estrogel, Euthyrox 100mg | postfrontal bilateral (500), parietal bilateral, precuneus, occ/temp bilateral | 6 | 5 |
| P05 | f | 61 | Alzheimers's Disease | 1 | Donepezil 10mg, **Escitalex 20mg**, Gingium 240mg, Memantin 20mg | frontal bilateral (500), parietal bilateral, precuneus, anterior cingulum, occ/temp bilateral | 9 | 7 |
| P06 | m | 72 | Alzheimers's Disease | 1 | Galantamin 16mg | frontal bilateral (600), parietal biltateral, precuneus, anterior cingulum | 14 | 3 |
| P07 | m | 67 | Alzheimers's Disease | 1 | Concor 5mg, Praluent 2x/month, Rivastigmin 9.5mg, Tamsulosin 0.4mg, Thrombo ASS 100mg | frontal bilateral (600), parietal bilateral, precuneus, anterior cingulum, occ/temp bilateral | 9 | 7 |
| P08 | m | 65 | Alzheimers's Disease | 1 | Rivastigmin 9.6mg | frontal bilateral (700), parietal ext bilateral, precuneus, anterior cingulum | 0 | 11 |
| P09 | f | 68 | Alzheimers's Disease | 1 | Donepezil 5mg, L-Thyroxin 50mg, Thrombo ASS 100mg | frontal ext. bilateral (700), parietal ext. bilateral, precuneus, cing cort, occ/temp bilateral | 1 | 2 |
| P10 | m | 65 | Alzheimers's Disease | 1 | **Escitalopram 5 mg**, Rivastigmin 9.5mg | frontal bilateral (600), parietal bilateral, cing cort ext, occ/temp bilateral | 12 | 10 |
| P11 | m | 52 | Alzheimers's Disease | 1 | Adjuvin 100mg, Donepezil 5mg | frontal bilateral (600), parietal bilateral, precuneus, occ/temp bilateral | 4 | 4 |
| P12 | f | 77 | Alzheimers's Disease | 1 | Thrombo ASS 100mg, Sortis 20mg, Euthyrox 75mcg, Rivastigmine 4,6mg/24 h, **Mirtabene 30mg** | frontal bilateral (700), partiel bilateral, precuneus, anterior cingulum | 29 | 5 |
| P13 | m | 80 | Alzheimers's Disease | 1 | Amaryl 3mg, Cerebokan 80mg, Ebixa 20mg, Jentadueto 2.5/1000mg, Thrombo ASS 100mg | frontal bilateral (400), parietal bilateral, precuneus ext, anterior cingulum, occ/temp bilateral | 0 | 0 |
| P14 | m | 82 | Alzheimers's Disease | 1 | Cerobokan 80mg, Enalapril 20mg, Exelon 9.5mg, Sortis 10mg, Thrombo ASS 100mg, Thyrex 75mg | frontal bilateral (600), parietal bilateral, precuneus, anterior cingulum, occ/temp bilateral | 3 | 2 |
| P15 | m | 56 | Alzheimers's Disease | 1 | Candesartan 16mg, Doxycyclin 200 | frontal bilateral (600), parietal bilateral, precuneus, anterior cingulum, occ/temp bilateral | 10 | 8 |
| P16 | m | 57 | Alzheimers's Disease | 1 | Donepezil 5mg | frontal bilateral (700), parietal bilateral, precuneus, anterior cingulum | 3 | 4 |
| P17 | m | 64 | Alzheimers's Disease | 1 | Tanganil 500 mg, MemoLoges/CogniLoges, Omega 3, Candesartan 16 mg (not constantly) | frontal ext. bilateral (700), parietal bilateral, precuneus, anterior cingulum, cerebral bilateral | 3 | 16 |
| P18 | f | 79 | Alzheimers's Disease | 1 | Aricept 10mg, **Mirtazapin 10mg**, Sifrol 0.35mg | frontal bilateral (700), parietal bilateral, precuneus, anterior cingulum, occ/temp bilateral | 1 | 4 |
| P19 | m | 61 | Alzheimers's Disease | 1 | Atorvastatin 40mg, Donepezil 5mg**, Duloxetin 60mg**, Euthyrox 150mg, Memantin 5mg, Selen Loges 200 NE, Vigantol 100iE Vit D3 | frontal bilateral (800), parietal bilateral, precuneus, anterior cingulum, occ/temp bilateral | 10 | 16 |
| P20 | f | 74 | Alzheimers's Disease | 1 | Atorvastatin 40mg, Calcimagon 1000mg, Donepezil 5-10 mg, Duspatalin, Euthyrox 125, Epril 10mg, Alnsoprazol 15mg | frontal bilateral (600), parietal bilateral, precuneus, anterior cingulum, occ/temp bilateral | 6 | 1 |
| P21 | f | 71 | Alzheimers's Disease | 1 | Clopidogrel 75mg, Donepezil 5mg, Rosuvastatin 5mg, Vitamin D3, Vitamin E | frontal bilateral (600), parietal bilateral, precuneus, anterior cingulum, occ/temp bilateral | 4 | 2 |
| P22 | m | 75 | Alzheimers's Disease | 1 | Aglandin 0.4mg, Euthyrox 75 mikrog, Rivsatigmin 13.3mg/h, **Wellbutrin 150mg** | frontal bilateral (600), parietal bilateral,precuneus, anterior cingulum, occ/temp bilateral | 17 | 20 |
| P23 | f | 67 | Alzheimers's Disease | 1 | Donepezil 5mg | DLPFC bilateral (600), parietal bilateral, precuneus, para/hippo/temp/occ bilateral | 10 | 7 |
| P24 | f | 66 | Alzheimers's Disease | 1 | Donepezil (discontinued during therapy pause), Memantin 29mg | frontal bilateral (600), parietal bilateral, precuneus, anterior cingulum, occ/temp bilateral | 1 | 5 |
| P25 | f | 57 | Alzheimers's Disease | 1 | Enalapril 20mg, Nevotens, Memando 20mg, Cadiopirin 100mg, Vitamin B12, Glukopadie 1000mg | frontal bilateral (600), parietal bilateral, precuneus, anterior cingulum, occ/temp bilateral | 2 | 0 |
| P26 | m | 65 | Alzheimers's Disease with early onset | 1 | Rivastigmin 4.6mg | frontal bilateral (500), parietal bilateral, precuneus, anterior cingulum, occ/temp bilateral | 4 | 0 |
| P27 | f | 65 | Atypical or mixed dementia | 1 | Blopress plus 8/12.5 (not constantly), Concor 5mg, Memantien 20mg, Metformin 1000mg, Pantoloc 20mg, Thrombo ASS 100mg, Zyprexa 2.5-5mg | frontal bilateral (700), parietal bilateral, precuneus, anterior cingulum | 0 | 0 |
| P28 | m | 75 | Dementia | 1 | Aricept 10mg, Cerebokan 80mg | frontal bilateral (600), parietal bilateral, precuneus, anterior cingulum, occ/temp bilateral | 5 | 2 |
| P29 | f | 78 | Dementia | 1 | Bisoprolol 5mg, Cal-D-Vita, Entresto 49mg/51 mg, Lasix 40 mg, Latanoprost 50 mg, Mesagran 3000mg, Pantoloc 40mg, Sortis 20mg, Spirono, Thrombo ASS 100mg | frontal ext bilateral (600), parietal bilateral, precuneus, anterior cingulum, occ/temp bilateral | 6 | 0 |
| P30 | f | 72 | Dementia | 1 | Dr.Böhm memory active capsules | frontal bilateral (500), parietal bilateral, precuneus, anterior cingulum, occ/temp bilateral | 2 | 2 |
| P31 | f | 81 | Dementia | 1 | Actavis, Cadesartan, Donepezil | frontal bilateral (700), parietal bilateral, precuneus, anterior cingulum, occ bilateral | 6 | 5 |
| P32 | m | 80 | Dementia | 1 | Amlodipin/Valsartan 80/5, Arosuva 20mg plus, Cerebokan 80mg, Donepezil 10mg, Exforge 160/5/12.5, Ezetimib, Oleovit D3, Pradaxa 150, **Sertralin 50mg** | frontal bilateral (500), parietal bilateral, precuneus, anterior cingulum, occ/temp bilateral | 9 | 8 |
| P33 | f | 63 | Dementia, PPA (Primary progressive aphasia) | 1 | Memantine 20mg, Oleovit D3, Cerebokan 80mg, Blopress PLUS 12,5mg**, Escitalopram 10mg**, Sitagliptin, 100mg | frontal bilateral (600), parietal bilateral, precuneus, anterior cingulum, temporal bilateral | 0 | 0 |
| P34 | m | 78 | Dementia-related development | 1 | Exelon 13.3mg/24h, Memantine 20mg | frontal bilateral (600), parietal bilateral, precuneus, anterior cingulum | 0 | 0 |
| P35 | m | 74 | Incipient dementia | 1 | Lovenox 40mg, Xarelto 20mg (paused) | frontal bilateral (700), parietal bilateral, precuneus, anterior cingulum, occ/temp bilateral | 1 | 1 |
| P36 | m | 82 | incipient sDAT | 1 | Donepezil 10mg, Nootropil, Tebofortan, Vitamin B | frontal bilateral (700), parietal ext bilateral, precuneus, anterior cingulum | 5 | 0 |
| P37 | m | 82 | Mild cognitive impairment | 1 | Androfin, Cerebokan, Omega 1400, Pro Macula, Rivastigmin, Thrombo ASS | frontal bilateral (700), parietal bilateral, precuneus, anterior cingulum, occ/temp bilateral | 7 | 9 |
| P38 | f | 81 | Mild cognitive impairment | 1 | Biopress 8-12mg, Concor 2.5mg, Diabetex 500mg, Donepezil 5mg, Lansobene 40mg, Thrombo ASS 100mg, Ursofalk 500mg | frontal bilateral (600), parietal bilateral, precuneus, anterior cingulum | 30 | 34 |
| P39 | f | 73 | Mild cognitive impairment | 1 | Amlodipin 5mg, Cerebokan 80mg, **Cipralex 10mg**, Eplerenon 25mg, Intuniv 2mg | frontal bilateral (700), parietal bilateral, precuneus, anterior cingulum | 9 | 9 |
| P40 | m | 76 | Mixed dementia | 1 | Aricept 5-10mg | frontal bilateral (700), parietal + motorcort bilateral, precuneus, anterior cingulum | 15 | 15 |
| P41 | m | 77 | Mixed dementia | 1 | Aricept 10mg, Atorvalan 20mg, Euthyrox 100mcg, Synjardy 12.5/850 mg, Thrombostad 100mg, Trental | frontal bilateral (600), parietal bilateral, precuneus, anterior cingulum, occ/temp bilateral | 3 | 7 |
| P42 | m | 82 | Mixed dementia | 1 | Bisoprolol 5mg, Candesartan 32mg, Jentadueto 2.5mg, Pioglitazon 15mg, Rivastigmin 9.5mg/24h, Visicare 5mg | frontal bilateral (500), parietal bilateral, precuneus, anterior cingulum, occ/temp bilateral | 35 | 29 |
| P43 | m | 82 | Mixed dementia | 1 | Memolan 10mg, Oleovit D3 | frontal bilateral (500), parietal bilateral, precuneus, anterior cingulum, occ/temp bilateral | 3 | 7 |
| P44 | m | 81 | Mixed dementia | 1 | Rivastigmin-patch 9.5 mg | frontal/DLPFC bilateral (600), parietal bilateral, precuneus, anterior cingulum, occ/temp bilateral | 13 | 12 |
| P45 | f | 78 | Mixed dementia, diabetes | 1 | Armolipid plus, Glyxambi 25/5mg, Mangsosolv, Nomexor 5mg, Oleovit D3, Spermedine memory, Tebonin 240mg | frontal bilateral (700), parietal bilateral, precuneus, anterior cingulum | 3 | 4 |
| P46 | m | 74 | Moderate dementia | 1 | Memolan 20mg, Paracetamol 500mg (long-term medication) | frontal bilateral (500), parietal bilateral, precuneus, anterior cingulum, occ/temp bilateral | 3 | 4 |
| P47 | m | 70 | Subjective cognitive impairment | 1 | Thrombo ASS 100mg, Aceclofenac Mite (not constantly) | frontal bilateral (600), parietal bilateral, precuneus, anterior cingulum | 3 | 0 |
| P48 | f | 81 | Senile dementia of the Alzheimer’s type | 1 | Calciduran Vit.D3 500mg/800 I.E, Donepezil 10mg, Inkontan 30mg, **Sertralin 100mg**, Zanipril | frontal bilateral (500), parietal ext bilateral, precuneus, anterior cingulum, occ/temp bilateral | 4 | 4 |
| P49 | m | 65 | Senile dementia of the Alzheimer’s type | 1 | Memantine 20mg, **Sertralin 100mg**, Thrombo ASS 100mg | frontal bilateral (600), centroparietal bilateral, precuneus ext, anterior cingulum | 6 | 3 |
| P50 | f | 78 | Senile dementia of the Alzheimer’s type | 1 | Acetylcys. Hex 600mg, Calciduran 500mg/800ie, Donepezil 10mg, Fentanyl hex 25mcg/h, Latanoprost 50mcg/ml, Neurobion, Ramipril 2.5mg, Tardyferon 80mg, Thrombo ASS 100mg, Vertirosan 50mg | frontal bilateral (700), parietal bilateral, precuneus, anterior cingulum | 8 | 10 |
| P51 | f | 77 | Semantic dementia | 1 | Calciduran 500mg/800mg, Cerebokan 80mg, **Escitalopram 15mg**, Exelon 13.3mg/24h, Inkontan 30mg, Lamotrigin 50mg, Metformin 1000mg, Thrombo ASS 100mg | frontal bilateral (500), parietal bilateral, precuneus, cing cort, left occ/tempt, cereb occ | 28 | 10 |
| P52 | f | 78 | Vascular dementia | 1 | Ezetim/simv 10/20mg, Motilium 10mg, Pantip 40mg, Rivastigmin 1.5mg, Tebefortan 40mg, Thrombo ASS 100mg | frontal bilateral (600), parietal bilateral, precuneus, anterior cingulum, occ/temp bilateral | 1 | 0 |
| P53 | f | 84 | Vascular dementia | 1 | Cerebokan, Thrombo Ass 100mg, Amlodilan 5mg, Exelon 20mg | frontal bilateral (600), parietal ext bilateral, precuneus, anterior cingulum, occ/temp bilateral | 9 | 3 |
| P54 | f | 75 | Vascular dementia | 1 | Bisoprolol 5mg, Cal-D-Vita, Entresto 49mg/51mg, Lasix 40mg, Latanoprost 50mg, Mesagran 3000mg, **Sertralin 50mg**, Sortis 20mg, Spirono, Thromo ASS 100mg | frontal bilateral (600), parietal bilateral, precuneus, anterior cingulum, temporal bilateral | 11 | 13 |
| P55 | m | 74 | Vascular dementia | 1 | Amlodipin 5mg, Blopress plus 16mg, Crestor 40mg, Fragmin 5000 units, lterium 1mg, Pantoloc 40mg, Thrombo ASS 100mg | DLPFC bilateral (700), centroparietal bilateral, precuneus ext., anterior cingulum ext. | 4 | 2 |
| P56 | m | 78 | Vascular dementia | 1 | Cerebokan 80mg, Clopidogrel 75mg, Donepezil 5mg, Gutron 5mg, Imurek, Mestinon 60mg, Oleovit D3, Quetialan 25mg, Rosamib 5/10mg | frontal bilateral (600), parietal bilateral, precuneus, anterior cingulum, occ/temp bilateral | 5 | 4 |
| P57 | f | 66 | Cerebellar ataxia (idiopathic late-onset cerebellar ataxia, ILOCA) | 2 | none | premotor cortex ext. bilateral including DLPFC bilateral (600), primary motor cortex bilateral, SMA/CMA, cerebellum bilateral | 11 | 16 |
| P58 | m | 65 | Iron deposition, PD | 2 | Pramipexol 1.05mg | premotor cortex ext. bilateral including DLPFC bilateral (800), primary motor cortex bilateral, SMA | 0 | 0 |
| P59 | f | 58 | Right-sided hemiparkinson | 2 | none | premotor cortex ext. bilateral including DLPFC bilateral (600), primary motor cortex bilateral, SMA/CMA | 5 | 2 |
| P60 | f | 73 | Idiopathic prakinson syndrome | 2 | Neupro 1mg, **Venlafab 75mg**, Quetiapin 25mg, Neuromultivit, Oleovit D3, Calciduran | premotor cortex ext. bilateral including DLPFC bilateral and Broca bilateral (900), centralregion bilateral, SMA/CMA | 12 | 9 |
| P61 | m | 69 | Idiopathic prakinson syndrome | 2 | Cerebokan 80mg, Madopar 100/25mg | frontal bilateral, parietal bilateral (500), centralregion bilateral, precuneus/midline, anterior cingulum | 4 | 6 |
| P62 | m | 76 | Parkinson's disease | 2 | Madopar 100/25mg, Pramipexol Retard 0.52/0.26mg, Rasagilin 1mg, Rivastigmin-patch 9.5mg | precentral region ext. bilateral including DLPFC bilateral (800), centralregion bilateral, SMA/CMA | 13 | 19 |
| P63 | m | 62 | Parkinson's disease | 2 | Cerebokan 80mg, Sifrol retard 2.1mg, Rasagilin Ari 1mg, Madopar Cr 100/25mg, Madopar 200/50mg | frontal bilateral (500), centralregion bilateral, precuneus, anterior cingulum | 27 | 14 |
| P64 | m | 73 | Parkinson's disease | 2 | Madopar 200/50mg, Madopar 100/25mg, Sifrol retard 3.15mg, Rasagilin 1mg, Pantoloc and NSAR and Replax (not constantly) | premotor cortex ext. bilateral including DLPFC bilateral (700), primary motor cortex bilateral, SMA/CMA | 3 | 0 |
| P65 | m | 73 | Parkinson's disease | 2 | Madopar 200/50mg, Madopar 100/25mg, Madopar 100/25mg (dispersible tablet), Sifrol retard 1.05mg, PK-Merz 100mg | premotor cortex ext. bilateral including DLPFC l (700), centralregion bilateral, SMA/CMA, anterior cingulum | 7 | 5 |
| P66 | m | 72 | Equivalence-type Parkinson’s disease | 2 | none | premotor cortex ext. bilateral including DLPFC bilateral (900), primary motor cortex bilateral, SMA/CMA | 11 | 5 |
| P67 | m | 48 | Parkinson’s disease of the hypokinetic-rigid predominant type | 2 | Rasagilin 1mg, Pramipexol retard 1.05/0.52mg, Levodopa 100mg | premotor cortex ext. bilateral including DLPFC bilateral and Broca bilateral (600), primary motor cortex bilateral, SMA/CMA | 7 | 5 |
| P68 | m | 66 | Multiple system atrophy | 2 | Candesartan 8mg | premotor cortex ext. bilateral including DLPFC bilateral (700), centralregion bilateral, SMA/CMA, cerebellum bilateral | 6 | 7 |
| P69 | m | 76 | Parkinson’s disease of the akinetic-rigid type | 2 | **Cipralex 10mg**, Rasagilin 1mg, PK-Merz, Corbilta 150, Thrombo ASS 100mg, Praxiten 15mg, Cerebokan 80mg, Mysoline**, Mirtazapin 30mg** | premotor cortex ext. bilateral including DLPFC bilateral (600), centralregion bilateral, precuneus, cingulate cortex | 6 | 9 |
| P70 | m | 58 | Parkinsonian syndrome | 2 | none | premotor cortex ext. bilateral including DLPFC bilateral (600), centralregion bilateral, SMA/CMA | 4 | 4 |
| P71 | m | 69 | Parkinsonian syndrome | 2 | Madopar 125mg, Ongentys 50mg | premotor cortex ext. bilateral including DLPFC bilateral (800), primary motor cortex bilateral, SMA/CMA | 2 | 1 |
| P72 | f | 71 | Parkinsonian syndrome | 2 | Neuropro 6mg/24 h, Rasagilin, Madopar 200/50mg | premotor cortex ext. bilateral including DLPFC bilateral (700), primary motor cortex bilateral, SMA/CMA | 4 | 2 |
| P73 | m | 76 | Parkinsonian syndrome | 2 | Madopar 100/25mg, Neuropro-patch 8mg, | prefrontal bilateral (700), centralregion bilateral, SMA/CMA | 0 | 2 |
| P74 | f | 84 | Parkinsonian syndrome | 2 | Madopar 100/25mg, Madopar Cr, Sifrol retard 3.15mg, Rasagilin 1mg, PK-Merz | premotor cortex ext. bilateral including DLPFC bilateral (800), centralregion bilateral, SMA/CMA | 2 | 2 |
| P75 | m | 65 | Parkinsonian syndrome | 2 | Madopar 50mg/12.5mg, Sifrol 1.05mg**, Cipralex 10mg**, Oleovit 12,5ml | premotor cortex ext. bilateral including DLPFC bilateral (700), centralregion bilateral, SMA/CMA | 0 | 1 |
| P76 | f | 73 | Progressive supranuclear palsy | 2 | Amantadin, Molaxole | premotor cortex ext. bilateral including DLPFC bilateral (600), centralregion bilateral, SMA ext., CMA ext. | 20 | 19 |
| P77 | m | 64 | Progressive supranuclear palsy | 2 | Levodopa/Benserazid 100/25mg, Levodopa/Benserazid retad 100/25mg, Atorvastatin 20mg, Tamsulosin retard 0.4mg, Artelac, Azilect 1mg | midline anterior including ACC* (1000 pulses), midline posterior, centralregion bilateral  *This is the only case without left DLPFC stimulation. Pulse count refers to ACC stimualtion | 32 | 8 |
| P78 | m | 66 | Progressive supranuclear palsy, Richardson syndrome | 2 | none | premotor cortex ext. bilateral including DLPFC bilateral (700), primary motor cortex ext. bilateral, precuneus, anterior cingulum ext. | 41 | 22 |
| P79 | f | 59 | Right-dominant Parkinson’s disease dementia | 2 | Requip Modutab 4mg, Rasagilin 1mg, Rivastigmin 1.5mg, Oxis Turbohaler 12 µg | frontal bilateral (500), partiel bilateral, precuneus, centralregion bilateral, SMA/CMA | 5 | 1 |
| P80 | m | 55 | Tremor | 2 | Amlodipin | premotor cortex ext. bilateral including DLPFC bilateral (900), centralregion bilateral, SMA/CMA | 0 | 0 |
| P81 | f | 49 | Apraxia | 3 | none | premotor cortex ext. bilateral including DLPFC bilateral (500), primary motor cortex bilateral, SMA/CMA, cerebral bilateral | 5 | 2 |
| P82 | f | 81 | Cerebral amyloid angiopathie | 3 | **Trittico 150mg**, Donepezil 5mg | frontal bilateral, parietal bilateral (500), precuneus, anterior cingulum, occ/temp bilateral | 2 | 4 |
| P83 | f | 28 | Memory impairment after anorexia | 3 | **Sertralin 100mg**, Aripiprazol 5mg | frontal ext. bilateral (700), parietal ext. bilateral, precuneus, anterior cingulum ext. | 31 | 22 |
| P84 | m | 81 | Global atrophy, aging brain, moderate microangiopathy | 3 | none | DLPFC etx. Bilateral (700), parietal bilateral, precuneus, anterior cingulum | 13 | 18 |
| P85 | f | 58 | Sequelae of cerebral ischemia | 3 | atorvaSTATin/Lipitor 80mg, Extracal, Colecalciferol 25mcg, Plavix/Clopidogrel 75mg, Vitamin B1, B6, B12 complex/Neurobion | DLPFC bilateral (500), parietal bilateral, precuneus, anterior cingulum, occ/temp bilateral | 28 | 6 |
| P86 | m | 64 | Progressive stroke | 3 | Lisinopril/Hydrochlorothiazide 20 mg/12.5 mg, **Duloxetin 60mg**, Thrombo ASS 100mg, Megnesium Verla, Lisinopril 10mg, **Mirtazapin Hexal 30mg** | DLPFC l (600), premotor cortex r, centralregion r, SMA/CMA, M1 foot area | 23 | 12 |
| P87 | m | 82 | Cryptogenic stroke | 3 | Rabeprazol 20mg, Ramipril/Hydrochlorothiazide 5/25mg, Ramipril 10mg, Zandidip 10mg, Sortis 80mg, Carvedilol 25mg, Ezetimib Sandoz 10mg, Vasonit retard 400mg, Plavix 75mg, Thrombo ASS 100mg | frontal bilateral (600), parietal adapted bilateral, precuneus, anterior cingulum, temporal bilateral | 11 | 4 |
| P88 | m | 80 | Vertigo, unspecified | 3 | none | frontal bilateral (600), parietal bilateral, precuneus, anterior cingulum | 3 | 2 |
